# Swi5-Sfr1 protein stimulates Rad51-mediated DNA strand exchange reaction through organization of DNA bases in the presynaptic filament

**DOI:** 10.1093/nar/gkt1257

**Published:** 2013-12-03

**Authors:** Louise H. Fornander, Axelle Renodon-Cornière, Naoyuki Kuwabara, Kentaro Ito, Yasuhiro Tsutsui, Toshiyuki Shimizu, Hiroshi Iwasaki, Bengt Nordén, Masayuki Takahashi

**Affiliations:** ^1^Department of Chemical and Biological Engineering, Chalmers University of Technology, S-41296 Gothenburg, Sweden, ^2^Research Unit FRE3478, Centre National de la Recherche Scientifique & University of Nantes, F-44322 Nantes cedex 3, France, ^3^Graduate School of Pharmaceutical Sciences, Faculty of Pharmaceutical Sciences, University of Tokyo, Tokyo 113-0033, Japan, ^4^Structural Biology Research Center, Photon Factory, Institute of Materials Structure Science, High Energy Accelerator Research Organization, KEK, Tsukuba, 305-0801, Japan and ^5^Department of Life Science, Graduate School of Bioscience & Biotechnology, Tokyo Institute of Technology, Yokohama 226-8501, Japan

## Abstract

The Swi5-Sfr1 heterodimer protein stimulates the Rad51-promoted DNA strand exchange reaction, a crucial step in homologous recombination. To clarify how this accessory protein acts on the strand exchange reaction, we have analyzed how the structure of the primary reaction intermediate, the Rad51/single-stranded DNA (ssDNA) complex filament formed in the presence of ATP, is affected by Swi5-Sfr1. Using flow linear dichroism spectroscopy, we observe that the nucleobases of the ssDNA are more perpendicularly aligned to the filament axis in the presence of Swi5-Sfr1, whereas the bases are more randomly oriented in the absence of Swi5-Sfr1. When using a modified version of the natural protein where the N-terminal part of Sfr1 is deleted, which has no affinity for DNA but maintained ability to stimulate the strand exchange reaction, we still observe the improved perpendicular DNA base orientation. This indicates that Swi5-Sfr1 exerts its activating effect through interaction with the Rad51 filament mainly and not with the DNA. We propose that the role of a coplanar alignment of nucleobases induced by Swi5-Sfr1 in the presynaptic Rad51/ssDNA complex is to facilitate the critical matching with an invading double-stranded DNA, hence stimulating the strand exchange reaction.

## INTRODUCTION

RAD51 protein (Rad51) is a eukaryotic ortholog of RecA recombinase that is highly conserved among different species (1,2). It catalyzes the strand exchange reaction, which is an essential step in homologous recombination. Rad51 is thus vital for cell survival and maintenance of the genomic information by assisting in mitosis in an error-free repair of double-strand breaks, lethal DNA damage (3,4), as well as creating gene diversity through involvement in DNA segregation in meiosis (5). However, too high or too low recombination activity appears to relate to cancer formation and cancer advancement (6,7), and the strand exchange activity of Rad51 is, therefore, tightly regulated in the cell (8). Interestingly, the intrinsic activity of Rad51 is much weaker than that of RecA, and it requires several accessory proteins for optimal activity both *in vitro* and *vivo* (9).

The Swi5-Sfr1 heterodimer protein is one such accessory protein (10–13). The protein was first discovered in fission yeast (*Schizosaccharomyces pombe*), but it has also been found in mammals where it exhibits similar activity (14). The protein is a heterodimer consisting of one Sfr1 protein and one Swi5 protein (12,15) bound non-covalently to each other by primarily two leucine zippers (16). The C-terminal domain of the Sfr1 binds to Swi5, whereas the N-terminal domain has affinity for Rad51 and DNA (15,16). The shape of Swi5-Sfr1, partially determined by X-ray crystallography (16), is long and somewhat crescent-shaped, making the protein fit well into the groove of the right Rad51-filament that surrounds the single-stranded DNA (ssDNA). Although a number of mediator proteins help Rad51 to nucleate onto RPA-covered ssDNA and form the primary reaction intermediate, Rad51/ssDNA/ATP filament, Swi5-Sfr1 apparently stimulates the strand exchange reaction by stabilizing this presynaptic filament (11,12,16). Despite extensive study, however, the exact stimulation mechanism is yet obscure.

It is admitted that Rad51, like RecA, catalyzes the strand exchange reaction by assembling onto ssDNA to form a helical filament and then promoting strand exchange with a second entering homologous DNA sequence (17). But, it is neither clear how the sequence comparison between the two DNA is made nor how the DNA strands are exchanged. Thus, clarifying the activation mechanism of Swi5-Sfr1 may also provide information about the mechanism of the strand exchange reaction itself.

For this purpose, we have examined the effect of Swi5-Sfr1 on the structure of the presynaptic filament, composed of fission yeast Rad51 (SpRad51), ssDNA and ATP, by flow linear dichroism (LD) spectroscopy. In flow LD, the complex filaments are aligned by hydrodynamic flow and the absorption difference of orthogonal forms of polarized light is measured (18). The signal is directly related to the local orientation of organized chromophores in the complex, in our case mainly the DNA bases and the tyrosine residues of the protein, relative to the filament axis. The LD technique also provides information about the degree of filament alignment in the hydrodynamic flow, which is related to the filament stiffness, length and shape. By this approach, we have previously studied the structure of Rad51/DNA as well as RecA/DNA complex filaments (19–21), and recently we have demonstrated that the DNA bases in the human Rad51 (HsRad51)/ssDNA/ATP filament are oriented nearly perpendicular to the filament axis in the presence of Ca^2+^, although almost no preferential orientation was observed in the presence of Mg^2+^ (22). This could be the reason for the higher strand exchange activity of HsRad51 in the presence of Ca^2+^ compared with Mg^2+^ (23). In the strand exchange reaction, base pairing probably takes place both during the search for homology, between the nucleobases of the ssDNA in the presynaptic filament and the nucleobases of the invading dsDNA, and during the strand exchange step. Local motions and random orientations of the bases in the ssDNA would certainly disfavor such pairing, whereas a perpendicular orientation of the bases would favor it, both kinetically and thermodynamically. EM and AFM studies of the triplex formed during homologous recombination support this hypothesis, as they show that the dsDNA and the ssDNA are wound coaxially (24,25).

In this study, we examine the Swi5-Sfr1-promoted structural changes of the SpRad51 presynaptic filament in the presence of Mg^2+^, which is the major divalent cation in the living cell (free Mg^2+^ 0.4–0.6 mM) (26). We can also correlate the structure of the nucleofilament to measured strand exchange activity of the filament. To compare with the strand exchange activation exerted by Ca^2+^, we also analyze the structure of the SpRad51/ssDNA/ATP presynaptic filament in the presence of Ca^2+^, although its cellular concentration is much less than that used in our experiments (a few millimolar compared with micro- to nanomolar range *in vivo*). Furthermore, to test if the effect of Ca^2+^ and that of Swi5-Sfr1 are synergetic, we measure the strand exchange activity and the flow LD of the presynaptic filament in the presence of Ca^2+^ and Swi5-Sfr1 in combination.

Our results indicate that the stimulatory effect on the SpRad51-promoted strand exchange reaction exerted by Swi5-Sfr1 is associated with a more perpendicular organization of the DNA bases in the presynaptic filament. Such a base-organization and correlated increase in strand exchange activity has previously been linked to the presence of Ca^2+^ (22,23), but we can now report that also the accessory protein Swi5-Sfr1, in the presence of the most abundant divalent cation *in vivo*, Mg^2+^, likely exerts its stimulatory effect by similar means for facilitating the base pairing with an incoming dsDNA.

## MATERIALS AND METHODS

### Materials

Fission yeast Rad51 (SpRad51) and fission yeast full-length Swi5-Sfr1 and Swi5-N-terminal-deleted Sfr1, Swi5-Sfr1C, (deletion of amino acids 1–180) proteins were purified as previously described (16). Poly(dT) was purchased from Sigma, and poly(dεA) was prepared by chemical modification of poly(dA) (Sigma) with chloroacetaldehyde (Aldrich) as described by Cazenave *et al.* (27). The concentration (in nucleobases) of poly(dT) was determined from UV absorption using the extinction coefficient ε_263nm _= 8520 M^−^^1^cm^−^^1^. The concentration and degree of modification of poly(dεA) were determined from the UV absorption spectrum using the formulas given by Ledneva *et al.* (28), providing ε_257nm_ = 3990 M^−^^1^ cm^−^^1^ and a modification degree of 93%. ATP was from Sigma.

### Flow LD measurements

LD was measured on a Chirascan spectropolarimeter (Applied Photophysics). The samples were aligned using an inner rotating Couette flow cell with a total path length of 1 mm, and, if not noted otherwise, with a shear gradient of 1250 s^−^^1^. The spectra were measured between 400 and 200 nm (bandwidth: 1 nm; data increment: 0.5 nm; time-per-point: 0.5 s) and four spectra were averaged to increase the signal to noise ratio. Baseline correction was made by subtracting a spectrum measured without rotation of the Couette flow cell. The complexes were formed by mixing 4 µM SpRad51 protein, 12 µM (base) poly(dT) or poly(dεA) and 300 µM ATP in the indicated buffer. As the LD signal became stable after ∼1 h incubation at room temperature and stayed unchanged for several hours, we present the spectra after 2 h incubation. The buffer used in the experiments, if not noted otherwise, contained 20 mM Tris–HCl (pH 7.5), 50 mM NaCl, 30% glycerol, 0.2 mM EDTA, 0.1 mM EGTA, 1.2 mM MgCl_2_ (or 1.2 mM CaCl_2_ when noted) and 0.3 mM ATP.

### Strand exchange activity

The strand exchange activity assay was performed as described by Nomme *et al.* (29), measuring the exchange between a single-stranded oligonucleotide of 58 bases and its homologous double-stranded oligonucleotide of 32 base pairs. Experiments were performed with 1.5 µM SpRad51, 20 nM (in fragment) ssDNA, 50 nM (in fragment) dsDNA, 1.2 mM MgCl_2_ (or CaCl_2_) in 20 mM Tris–HCl (pH 8), 50 mM NaCl, 1 mM ATP, 1 mM DTT, 100 µg/ml BSA, 2% glycerol, 0.0075% Tween 20 (Sigma) and indicated concentration of Swi5-Sfr1.

## RESULTS AND DISCUSSION

### Swi5-Sfr1-promoted structural changes of SpRad51 presynaptic filaments

**Figure 1.**
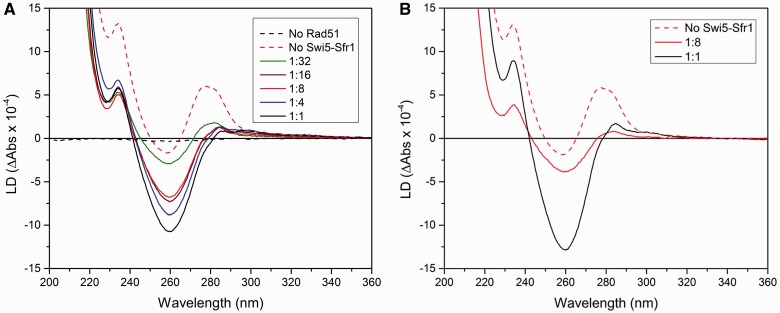
LD spectra showing structural changes of SpRad51/poly(dT) filament induced by Swi5-Sfr1. (**A**) LD spectra of SpRad51/poly(dT) complex filaments formed by mixing 4 mM SpRad51 and 12 mM poly(dT), ratios Swi5-Sfr1 to SpRad51 are indicated. The LD spectrum of poly(dT) with only Swi5-Sfr1 is also shown (black dashes). (**B**) Corresponding experiments performed using Swi5-Sfr1C instead of full length Swi5-Sfr1.

We investigate the effect that Swi5-Sfr1 protein exerts on the structure of the presynaptic filament of SpRad51 by flow LD. LD is the differential absorption of parallel and perpendicularly polarized light, LD = A_||_ − A_⊥_ (18)_,_ and it reflects the orientation of chromophores, such as the aromatic nucleobases of DNA and the tyrosine residues of protein, relative to the filament orientation axis. We measure LD spectra of ATP/SpRad51/ssDNA complexes formed in the presence of Mg^2+^, the major divalent ion in the cell. As ssDNA, we first used poly(dT) because it has neither particular secondary structure nor ability to form hairpins, and serves, therefore, as a good standard model for ssDNA. Neither poly(dT) nor SpRad51, due to flexibility and small size, respectively, gives rise to any significant LD signal on their own (results not shown). By contrast, the SpRad51/poly(dT) complexes exhibit strong LD signals both in the presence and absence of Swi5-Sfr1 (Figure 1A). This observation confirms that SpRad51 forms regular and stiff filaments around poly(dT). The concentration of ATP was high enough (0.3 mM) to minimize the effect of ADP accumulation on ATP hydrolysis by SpRad51. The LD spectra remained unchanged for several hours.

The LD spectrum of SpRad51/poly(dT) without Swi5-Sfr1 exhibits a positive band at 280 nm, a negative band around 260 nm and a large positive signal <220 nm with a small peak ∼230 nm (red dashes Figure 1A). The spectrum with SpRad51 is similar to that of HsRad51/poly(dT) complex with ATP/Mg^2+^, which has previously been examined by LD and molecular modeling combined (22). Both filaments demonstrate weak intensity of the negative LD signal at 260 nm relative to the positive signal at 280 nm. In our earlier study, we could conclude that the DNA bases in this nucleofilament with HsRad51 have no particular orientation (22) and, as both filaments display such high similarity in their LD spectra, we suggest that the bases in the filament formed with SpRad51 lack orientation as well. The negative signal at 260 nm is probably related to the ATP, whereas the positive band ∼280 nm arises from the tyrosine residues in the protein (22). There is a small variation at 230 nm in the LD spectrum with SpRad51 compared with that with HsRad51: a weak peak followed by a trough is seen at this wavelength in the LD spectrum for the SpRad51 filament, although these features are absent in the spectrum for the HsRad51 filament (22). This difference probably originates from deviations in organization of tyrosines, which have strong absorption at 227 and 278 nm. SpRad51 contains one more tyrosine than HsRad51: 11 compared with 10, out of which only 6 residues are conserved in common (2).

Addition of Swi5-Sfr1 strongly affects the LD spectrum (continuous line spectra in Figure 1A). The negative band ∼260 nm increases in intensity relative to the positive band at 280 nm, and the spectral shape of SpRad51/poly(dT) in complex with Swi5-Sfr1 is similar to that of the HsRad51/poly(dT) filament formed in the presence of ATP/Ca^2+^ (22). We have shown that this large negative band at 260 nm for HsRad51/poly(dT)/ATP/Ca^2+^ stems from a preferentially perpendicular orientation of the DNA bases. As Swi5-Sfr1 alone can also bind to DNA (16), we verified that the observed spectral changes are not due to Swi5-Sfr1 binding directly to DNA by measuring the LD spectrum of poly(dT) with only Swi5-Sfr1 (no SpRad51) (black dashed spectrum in Figure 1A). The mixture exhibited only a weak signal, confirming that the large change in the LD signal originates from structural rearrangement of the SpRad51/ssDNA filament when Swi5-Sfr1 is added.

To test whether there is any correlation between the detected structural changes of the Rad51-DNA filament on addition of Swi5-Sfr1 and the Swi5-Sfr1-dependent activation of the strand exchange reaction, we measured LD of the filament at various Swi5-Sfr1/SpRad51 ratios and compared the received spectral concentration profile with the increase in strand exchange activity on Swi5-Sfr1 addition. It has been reported that the maximum stimulation of strand exchange by Swi5-Sfr1 occurs at ∼1 molecule of Swi5-Sfr1 per 10–20 SpRad51 (16). By LD, we observe that the SpRad51/DNA structure starts to alter at ratio 1:32 (Swi5-Sfr1 relative to SpRad51) (Figure 1A). When adding more Swi5-Sfr1, the LD signal continues to alter, but the change saturates at ratio 1:16. When increasing the concentration to 1:4 and above, we detect some further change in LD signal. Therefore, in the subsequent LD experiments, we used the ratio 1:8, as this ratio appears to saturate the Rad51-DNA filament. As the structural changes observed by LD are nicely correlated with the stimulation of the strand exchange reaction, we conclude that the increase in strand exchange activity on addition of Swi5-Sfr1 is due to structural changes of the nucleofilament.

### Swi5-Sfr1 promotes structural changes by its binding to the Rad51 filament

To get a better understanding of the Swi5-Sfr1 binding to the SpRad51/DNA filament, we measured LD of the nucleofilament in the presence of Swi5-Sfr1C. In Swi5-Sfr1C, the N-terminal domain (1–180 amino acids) of Sfr1 has been deleted, and therefore the protein does not possess DNA binding capacity. However, it can still stimulate the strand exchange activity although a higher amount of Swi5-Sfr1C is needed than Swi5-Sfr1 (16). We observe that the change in LD signal promoted by Swi5-Sfr1C when binding to the SpRad51/poly(dT) complex is similar to that by full length Swi5-Sfr1 (Figure 1B). We, therefore, conclude that the structural changes induced by Swi5-Sfr1 on the SpRad51/DNA filament, and the associated increase in strand exchange activity, do not require that Swi5-Sfr1 comes in direct contact with the DNA.

A larger amount of Swi5-Sfr1C was required to promote a corresponding structural changes as with Swi5-Sfr1 on the SpRad51/poly(dT) filament, which is in agreement with the previously observed smaller stimulation efficiency of the truncated protein (16). The ratio 1:8 Swi5-Sfr1C to SpRad51, an amount that gave a ‘complete change’ for the full length Swi5-Sfr1, does not promote any large structural change (Figure 1B). Instead, the Swi5-Sfr1C concentration had to be increased to 1:1 to achieve the corresponding change in LD signal. Our previous results showed that the maximum stimulation activity required 1:1 ratio of Swi5-Sfr1C (16), and we can thus confirm that the structural changes are related to the stimulation of strand exchange reaction. The N-terminal domain of Sfr1 is believed to bind first to the nucleofilament, and through its binding facilitating the anchoring of the Sfr1 C-terminal domain in complex with Swi5 (16). The deletion of the N-terminal of Sfr1 might decrease the affinity of the heteroprotein for the Rad51-DNA filament and, therefore, a higher concentration, as shown here, of Swi5-Sfr1C is needed to produce the same effect as full length Swi5-Sfr1.

### Swi5-Sfr1 promotes strand exchange through perpendicular organization of the DNA bases

**Figure 2.**
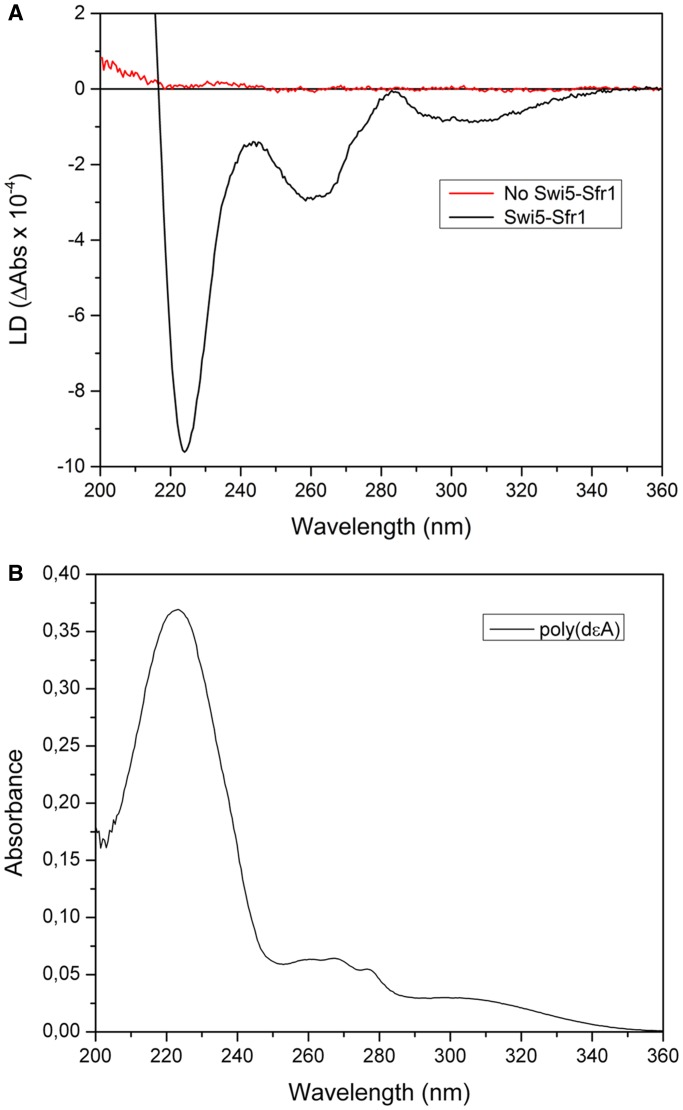
LD measurements using poly(dA) analog, poly(dεA), to demonstrate perpendicularly orientation of nucleobases in the presence of Swi5-Sfr1. (**A**) LD of SpRad51/poly(dεA)/ATP/Mg^2+^ filaments with and without Swi5-Sfr1 (Swi5-Sfr1 to SpRad51 ratio 1:8). Higher shear gradient (3125 s^−1^ versus 1250 s^−1^) was applied to amplify the weak LD signal of SpRad51/poly(dεA) filament without Swi5-Sfr1. (**B**) Absorption spectrum for poly(dεA).

To more clearly assess the orientation of the DNA bases, in particular in the presynaptic filament with Swi5-Sfr1, we measured the LD spectra of SpRad51 filaments with poly(dεA) as replacement for poly(dT). In poly(dεA), the adenine bases of poly(dA) are modified to 1,N6-ethenoadenine (εA), which has an additional absorption band centered at 310 nm (Figure 2B) (30), where no other chromophore of the SpRad51/DNA/ATP complex absorbs. All absorption bands are due to transition moments polarized in the plane of the chromophore (30), and are therefore expected to yield negative LD for perpendicular base orientation. Also importantly, the LD signal >300 nm is unambiguously related to the orientation of the bases within the nucleoprotein filament.

The LD spectrum of SpRad51/poly(dεA) without Swi5-Sfr1 exhibits only weak LD signal (red curve, Figure 2A), probably due to that the poly(dεA) is short compared with poly(dT) and that SpRad51 has weaker affinity to poly(dεA) than poly(dT). We still managed to obtain a significant signal by increasing the shear flow (from 1250 s^−^^1^ to 3125 s^−^^1^). The SpRad51/poly(dεA) filament exhibits a positive band ∼280 nm, a negative band at 260 nm and a large positive band at 210 nm with a peak and trough feature ∼230 nm. The shape of the spectrum is thus similar to that obtained with poly(dT) (see Figure 1A). This observation, along with the absence of any significant signal ∼310 nm, indicates complete absence of LD signal from DNA. We can now unambiguously conclude that, in the absence of Swi5-Sfr1, the DNA bases in the SpRad51 filament are not oriented in an organized manner, just as we have also observed previously for HsRad51 (22).

The addition of Swi5-Sfr1 strongly modifies the shape of the LD spectrum (black curve, Figure 2A). First, we can see a negative band appearing at 310 nm demonstrating a preferentially perpendicular orientation of the nucleobases of poly(dεA). Besides the signal at 310 nm, the other absorption bands of poly(dεA) (Figure 2B), at 274 and 238 nm (30), are also negative. The relative intensities of LD signals at those wavelengths are approximately identical to those of the corresponding absorption bands of poly(dεA), indicating the same angles of the different transition moments relative to the filament axis, as expected for a perpendicular orientation of DNA bases (all angles near 90°). This conclusion follows from the relation between angle, α, of the transition moment relative to the fiber axis and the ratio of LD to absorbance A_iso_ for the unoriented sample: LD/A_iso_ = (3/2) S (3 <cos^2^α>-1) where S is a parameter describing the degree of orientation (18). For example, with a strongly tilted nucleobase plane different transitions will appear with different angles α and, hence, different LD/A_iso_ ratios. However, the negative band at 260 nm in the LD spectrum stems from the orientation of adenine in ATP in the complex. Arguments for this conclusion are that there is no clear absorption peak at 260 nm in poly(dεA), and that the 260 nm LD peak is also visible without Swi5-Sfr1, when there is no orientation of the DNA bases. Thus, the orientation of the ATP is not markedly modified on binding of Swi5-Sfr1. We also note that the SpRad51/poly(dεA) filament in the presence of Swi5-Sfr1 exhibits a much larger LD signal than in its absence, even at mild shear velocities (1250 s^−^^1^), showing that the binding of Swi5-Sfr1 indeed significantly stiffens the Rad51 filament.

The Rad51 protein has previously been shown to create ‘filament patches’ on the DNA, consisting of clusters of a few tens of monomers (31). The Rad51 protein may nucleate at multiple sites on the DNA, creating several small filament patches with small gaps in between (32). The gaps are a result of adjacent patches being out of phase relative to each other, either because of opposite binding polarities (in case of ssDNA) or due to that there are insufficient space in between two filaments, which would be the case with only one or two bases (or base pairs) in between. The uncovered DNA between two adjacent filament patches may behave as hinges, making the whole Rad51/DNA complex more flexible despite many rigid patches. Compared with its bacterial homolog RecA, Rad51 demonstrates a rather low binding cooperativity (31), and therefore it does not form as long or as complete filaments on the DNA (33). The increase in orientation and rigidity that we observe when Swi5-Sfr1 binds to the SpRad51/poly(dεA) filament might also have a contribution from increased stiffness as adjacent Rad51 patches become ‘bridged’ by Swi5-Sfr1 binding in the groove of the Rad51 filament, a possibility we cannot exclude.

### The structural changes promoted by Swi5-Sfr1 and Ca^2+^ are similar and non-additive

The presence of Ca^2+^ stimulates the strand exchange activity of HsRad51 (23) which we, in a previous study, proposed to be related to a perpendicular orientation of the DNA bases in the HsRad51/ssDNA filament. Such an orientation effect was not observed for the HsRad51/ssDNA filament in the presence of Mg^2+^ (22).

**Figure 3.**
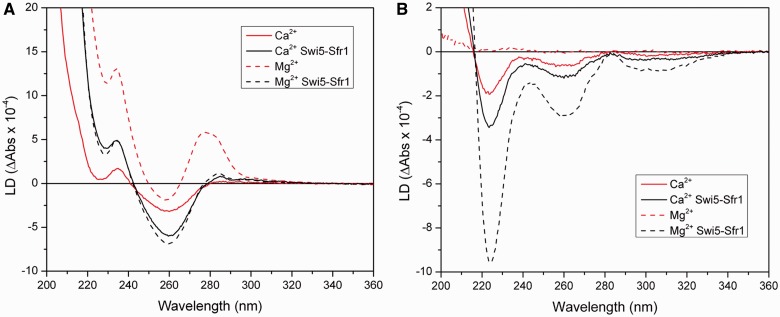
LD spectra demonstrating that Swi5-Sfr1 exerts different structural changes on presynaptic filament depending on the presence of Mg^2+^ or Ca^2+^. (**A**) LD spectra of SpRad51/poly(dT) complexes formed with and without Swi5-Sfr1 (ratio 1:8 of Swi5-Sfr1 relative to SpRad51) in the presence of Ca^2+^ (continuous line). The presence of Mg^2+^ is inserted as a reference (dashed line). (**B**) LD spectra for the same experiment but with poly(dεA) instead of poly(dT).

Here we have compared the effect that Swi5-Sfr1 exerts on the structure of the presynaptic filament and on the strand exchange activity with the stimulatory effect promoted by Ca^2+^. We first analyzed the LD spectrum of the SpRad51/poly(dT) filament formed in the presence of Ca^2+^. The spectrum shows a large negative LD band at 260 nm that indicates preferentially perpendicular orientation of DNA bases (Figure 3A), and it is similar to that obtained with Swi5-Sfr1 in the presence of Mg^2+^ (see Figure 1A). However, the intensity is about half of that with Swi5-Sfr1 and Mg^2+^, suggesting that the filament is significantly less rigid. Adding Swi5-Sfr1 to the SpRad51/poly(dT)/Ca^2+^ filament increases the intensity of the LD spectrum without changing the spectral shape, indicating that it is mostly the stiffness and not the structure of the filament that is altered. We verified that the addition of Swi5-Sfr1 to poly(dT) alone does not promote the development of any significant LD signal, not even in the presence of Ca^2+^ (results not shown). Thus, the change in LD can be ascribed as entirely due to the binding of Swi5-Sfr1 to the SpRad51-DNA filament. Similar results were obtained with poly(dεA) (Figure 3B). To conclude, Swi5-Sfr1 promotes similar structural changes in the presynaptic filament as Ca^2+^, but Swi5-Sfr1 makes the filament more rigid than does Ca^2+^.

Related to this finding, Nimonkar *et al.* recently published results about the stimulation of Dmc1 by Tid1 protein, a Rad54 paralog, during genetic recombination in *Saccharomyces cerevisiae* (34). They showed that the Tid1 protein stabilizes the presynaptic Dmc1-ssDNA filaments to the same form that is also induced by the presence of Ca^2+^. They proposed that Tid1 is the physiological inducer of a stable and active Dmc1-ssDNA presynaptic filaments, this since the Ca^2+^ concentration *in vivo* is too low, even during meiosis (35). In conclusion, it is interesting that the eukaryotic recombination proteins could be stimulated by an accessory protein mimicking Ca^2+^.

**Figure 4.**
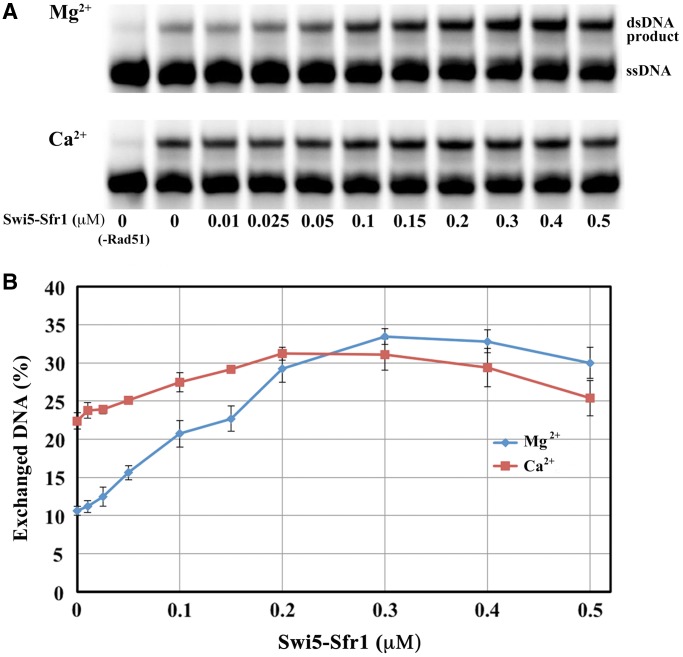
Swi5-Sfr1 and Swi5-Sfr1C affect the DNA strand exchange activity of SpRad51. SpRad51-promoted strand exchange between labeled ssDNA and its homologous dsDNA in the presence of Mg^2+^ or Ca^2+^, with concentration of Swi5-Sfr1 as indicated. Experiments performed as described in text.

We also examined whether the addition of Swi5-Sfr1 to filament formed in the presence of Ca^2+^ may stimulate the strand exchange reaction further. In the absence of Swi5-Sfr1, SpRad51 exhibits about twice higher strand exchange activity in the presence of Ca^2+^ than in the presence of Mg^2+^, showing that Ca^2+^ exerts its stimulatory effect also on SpRad51 (Figure 4), as observed for HsRad51 (22,23). Addition of Swi5-Sfr1 resulted in a large stimulatory effect for Mg^2+^, but only a small further increase with Ca^2+^ (3.4 times compared to 1.4 times) (Figure 4). Interestingly, the maximum strand exchange activity in the presence of Swi5-Sfr1 is similar for both cations. We can thus conclude that the effect of Ca^2+^ is not additive to that of Swi5-Sfr1, suggesting that Ca^2+^ and Swi5-Sfr1 stimulate the strand exchange reaction in similar ways, most likely via the mechanism of perpendicular orientation of DNA bases in the presynaptic filament. We note that the effect on the strand exchange activity exerted by Swi5-Sfr1 is larger than that of Ca^2+^ alone. This is probably due to the fact that Swi5-Sfr1 not only orients the nucleobases, seen from the LD experiments, but also may stiffen the Rad51 filament, which could further help to facilitate the strand exchange reaction.

By strand exchange assay, we tried to verify that the Swi5-Sfr1 protein exerts a stabilizing effect on the presynaptic filament. In the absence of Swi5-Sfr1, the strand exchange activity was observed to level out after 15 min for both Ca^2+^ and Mg^2+^. By contrast, when Swi5-Sfr1 was present the reaction continued for ∼60 min, which would be consistent with the formation of a more stable presynaptic filament (Supplementary Figure S1). We also performed strand exchange assay with the N-terminal deleted Swi5-Sfr1, Swi5-Sfr1C, and obtained similar results as with the wild-type protein (Supplementary Figure S2). Again, a larger amount of Swi5-Sfr1C was required for stimulation, as also observed in previous experiments (16) and in correlation with our LD experiments. Although these results could suggest that Swi5-Sfr1C may stimulate the reaction in a similar manner as the full length Swi5-Sfr1, they must be taken with great care as also variation with length of DNA could influence (*vide infra*).

We note that SpRad51 exhibits weaker strand exchange activity than HsRad51. To observe significant strand exchange activity we, therefore, used three times higher protein concentration than in our previous study with HsRad51 (22). The maximum stimulation occurs at ∼1 Swi5-Sfr1 per 5 SpRad51, whereas a smaller amount of Swi5-Sfr1 (1 per 10–20) is required for maximum stimulation in case of strand exchange with longer DNA (16). The structure and form of the long and crescent-shaped Swi5-Sfr1 may suggest that one molecule of Swi5-Sfr1 could form contacts with some 10∼20 SpRad51 molecules on the presynaptic SpRad51-DNA filament (15,16). For electrostatic reasons, it is reasonable that Swi5-Sfr1 might bind less well to filaments formed on short oligonucleotides, such as those used in our strand exchange assay, where a maximum of 19 SpRad51 molecules are able of binding to each DNA oligonucleotide. This might be the reason why we require a larger amount of Swi5-Sfr1 to promote the strand exchange, and also why our recorded strand exchange activity is somewhat lower compared to that with longer DNA (16).

## CONCLUSIONS

Our LD data suggest that Swi5-Sfr1 may stimulate the catalytic function of the SpRad51-DNA presynaptic filament by promoting a preferentially perpendicular orientation of DNA bases, and potentially also by causing an increase in the stiffness of the presynaptic filament. Along with the fact that Ca^2+^, as another activating element, also promotes perpendicular orientation of DNA bases, our results emphasize the importance of DNA base orientation for the strand exchange reaction. With Mg^2+^, which is the most abundant cation *in vivo*, no perpendicular base orientation is detected until Swi5-Sfr1 is added. The induced perpendicular alignment of DNA bases in the presynaptic Rad51/ssDNA complex, we propose, facilitates the matching with the bases of an invading double-stranded DNA through their coplanar orientation both in kinetic and thermodynamic terms by favoring the reaction entropy.

## SUPPLEMENTARY DATA

Supplementary Data are available at NAR Online.

Supplementary Data
