# Supplementary material for: Swi5-Sfr1 protein stimulates Rad51-mediated DNA strand exchange reaction through organization of DNA bases in the presynaptic filament
Source: Nucleic Acids Res. 2013 Dec 3;42(4):2358–65. doi: 10.1093/nar/gkt1257 (PMC3936755; doi:10.1093/nar/gkt1257)
Supplement: Supplementary Data [file supp_42_4_2358__index.html]

Supplementary Data 

# Swi5-Sfr1 protein stimulates Rad51-mediated DNA strand exchange reaction through organization of DNA bases in the presynaptic filament

## Supplementary Data

files

**Files in this Data Supplement:**

- Supplementary Data - pdf file
