# Supplementary material for: Swi5-Sfr1 protein stimulates Rad51-mediated DNA strand exchange reaction through organization of DNA bases in the presynaptic filament
Source: Nucleic Acids Res. 2013 Dec 3;42(4):2358–65. doi: 10.1093/nar/gkt1257 (PMC3936755; doi:10.1093/nar/gkt1257)
Supplement: Supplementary Data [file supp_gkt1257_nar-02030-d-2013-File007.pdf]

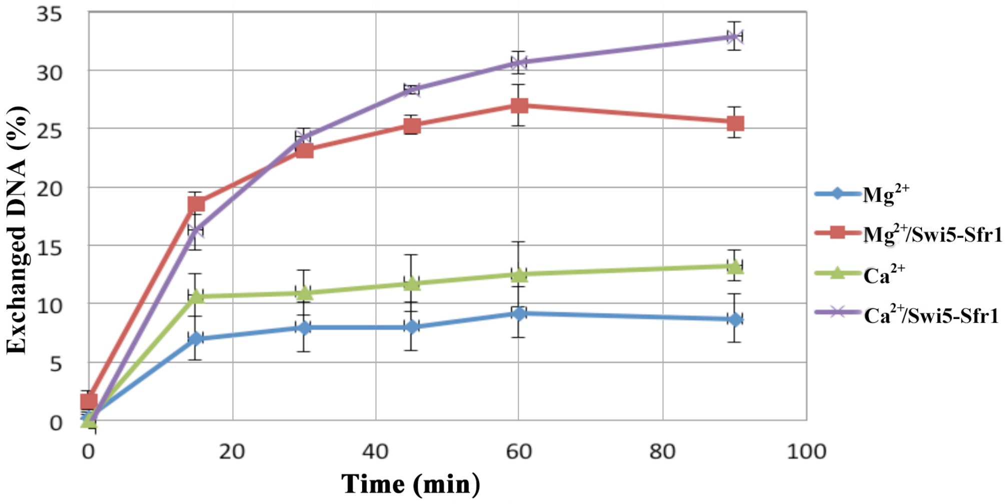

Supplementary Figure 1. Effect of incubation time in the strand-exchange assay of SpRad51/Swi5-Sfr1 with Ca^{2+} or Mg^{2+}. The experiments were performed as described in Material & Methods, but with the exception that the 1 hour incubation time was altered. The concentration of Swi5-Sfr1 was 0.2 μM.

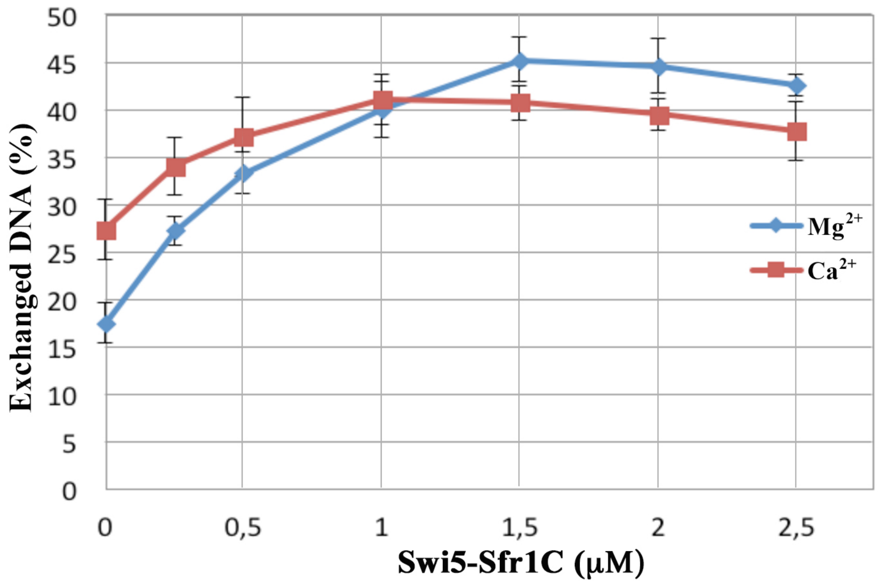

Supplementary Figure 2. Effect of Swi5-Sfr1C on the strand-exchange activity of SpRad51. The experiments were performed as described in Material & Methods. The effect of Swi5-Sfr1C is similar to that of the wild-type protein, but a larger amount of Swi5-Sfr1C had to be used.
